# Intra-amniotic infection with *Ureaplasma parvum* causes serovar-dependent white matter damage in preterm fetal sheep

**DOI:** 10.1093/braincomms/fcaf182

**Published:** 2025-05-24

**Authors:** Dima Abdu, Graeme R Polglase, Sharmony B Kelly, Sinead Murphy, Ilias Nitsos, Claudia A Nold-Petry, Suhas G Kallapur, Alan H Jobe, John P Newnham, Timothy J Moss, Robert Galinsky

**Affiliations:** The Ritchie Centre, Hudson Institute of Medical Research, Melbourne 3168, Victoria, Australia; Department of Obstetrics and Gynaecology, Monash University, Melbourne 3800, Victoria, Australia; The Ritchie Centre, Hudson Institute of Medical Research, Melbourne 3168, Victoria, Australia; Department of Paediatrics, Monash University, Melbourne 3800, Victoria, Australia; The Ritchie Centre, Hudson Institute of Medical Research, Melbourne 3168, Victoria, Australia; Department of Obstetrics and Gynaecology, Monash University, Melbourne 3800, Victoria, Australia; Department of Obstetrics and Gynaecology, Monash University, Melbourne 3800, Victoria, Australia; The Ritchie Centre, Hudson Institute of Medical Research, Melbourne 3168, Victoria, Australia; The Ritchie Centre, Hudson Institute of Medical Research, Melbourne 3168, Victoria, Australia; Department of Paediatrics, Monash University, Melbourne 3800, Victoria, Australia; Department of Pediatrics/Neonatology, UCLA Mattel Children’s Hospital, Los Angeles, CA 90095, USA; Division of Neonatology, Cincinnati Children’s Hospital Research Foundation, Cincinnati, OH 45229-3026, USA; University of Cincinnati College of Medicine, Cincinnati, OH 45267, USA; Division of Obstetrics and Gynaecology, Medical School, The University of Western Australia, Perth 6009, Western Australia, Australia; The Ritchie Centre, Hudson Institute of Medical Research, Melbourne 3168, Victoria, Australia; Department of Obstetrics and Gynaecology, Monash University, Melbourne 3800, Victoria, Australia; The Ritchie Centre, Hudson Institute of Medical Research, Melbourne 3168, Victoria, Australia; Department of Obstetrics and Gynaecology, Monash University, Melbourne 3800, Victoria, Australia

**Keywords:** white matter injury, chorioamnionitis, Ureaplasma, infection, preterm brain injury

## Abstract

*Ureaplasma parvum* is commonly isolated from the amniotic fluid of pregnancies complicated by infection. While some studies have shown an association between intra-amniotic *Ureaplasma* species infection and brain injury and/or adverse neurodevelopment, others have not. The relationship between antenatal exposure to microbial infection and risk of poor neurological outcome is complex and multifactorial and may reflect diversities in microbial pathogenicity along with the duration and severity of the fetal inflammatory response to microbial infection. This study aimed to determine the impact of chronic intra-amniotic infection with *Ureaplasma parvum* serovars 3 and 6, which are among the most common serovars isolated in pregnancies complicated by infection/inflammation, on white matter development in preterm fetal sheep. Pregnant ewes carrying singleton or twin fetuses (55 days gestational age, term = 145 days) were randomly allocated to receive an ultrasound-guided intra-amniotic injection of *Ureaplasma parvum* serovars 3 (*n* = 11), 6 (*n* = 16) or media (control, *n* = 6). At 125 days of gestation, the ewe and foetus were euthanized and the fetal brain was collected for immunohistochemistry. Total numbers of oligodendrocytes (oligodendrocyte transcription factor 2-positive cells) in the periventricular white matter tract were higher in *Ureaplasma parvum* serovar 6-exposed fetuses than control. Numbers of mature oligodendrocytes [anti-adenomatous polyposis coli clone (CC) 1-positive cells] and myelin density (% area fraction of myelin basic protein-positive) in the periventricular and intragyral white matter tracts were lower in *Ureaplasma parvum* serovar 6-exposed fetuses than control. Myelin anisotropy was lower in serovar 6-exposed fetuses than control. There were no differences in numbers of total or mature oligodendrocytes, myelin density and anisotropy in *Ureaplasma parvum* serovar-3-exposed fetuses compared to control. Cell death, numbers of neurons, total and reactive (signal transducer and activator of transcription 3-positive) microglia and astrocytes did not differ between *Ureaplasma parvum*-exposed fetuses and controls within the premotor cortex and striatum. Chronic intra-amniotic infection with *Ureaplasma parvum* serovar 6, but not 3, impaired oligodendrocyte maturation and myelination within the large white matter tracts of the preterm sheep brain. These data suggest that the impact *Ureaplasma parvum* infection on white matter development may be serovar dependant, which may help to explain why some fetuses exposed to intra-amniotic *Ureaplasma* infection have adverse neurodevelopmental outcomes while others do not. Overall, this study demonstrates that greater emphasis needs to be placed on the taxonomy of *Ureaplasma* infection when designing and interpreting clinical and preclinical studies of fetal infection and neurodevelopmental outcomes.

## Teaser

Abdu *et al*. reports that intra-amniotic infection with *Ureaplasma parvum* impairs myelination in the preterm brain and the impact of *U. parvum* infection on white matter development may be serovar-dependent. This may explain why some fetuses exposed to intra-amniotic Ureaplasma infection have adverse neurodevelopmental outcomes while others do not.

## Graphical Abstract

**Graphical Abstract:**
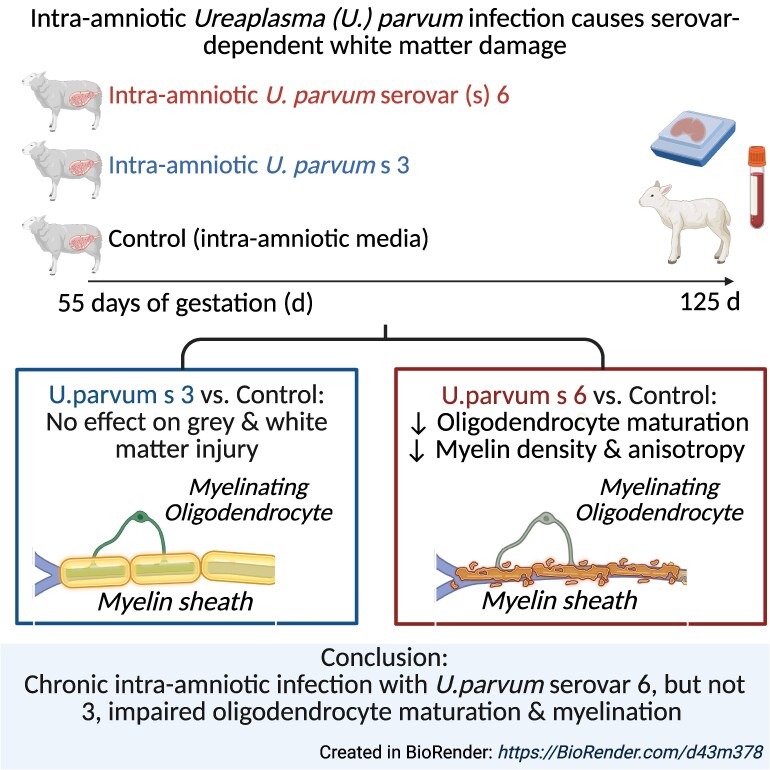

## Introduction

Intrauterine infection/inflammation, which often manifests as chorioamnionitis, is commonly associated with adverse neurological outcomes, such as cerebral palsy.^1^  *Ureaplasma* spp. are among the most common microorganisms isolated from the amniotic cavity in cases of intrauterine infection/inflammation.^2^ The presence of *Ureaplasma* spp. in amniotic fluid is associated with an increased risk of adverse psychomotor development, intraventricular haemorrhage and cerebral palsy at 2 years of age.^3^

Ureaplasmas are classified into two biovars, *Ureaplasma urealyticum* and *U. parvum*, based on their genetic and phenotypic differences.^4^  *Ureaplasma parvum* is more prevalent than *U. urealyticum*, with a higher colonization rate in the vaginal microbiome of childbearing adult females.^4-7^ Both biovars are detected in intra-amniotic infections; however, *U. parvum* serovars 3 and 6 are among the most common ureaplasmas isolated from amniotic fluid.^8,9^  *Ureaplasma parvum* serovar 3 is the most common serovar detected in pregnant and non-pregnant females, and urogenital colonization has been linked to reduced fertility in males and females.^10,11^  *Ureaplasma parvum* serovar 6 was the most common serovar isolated from pregnant females who delivered preterm^12^ and has strong adherence to epithelial cells and sperm,^13^ suggesting that it may be more pathogenic.

While some studies have shown an association between intra-amniotic *Ureaplasma* spp. infection and brain injury and/or adverse neurodevelopment, others have not.^3,14^ The relationship between antenatal exposure to microbial infection and risk of poor neurological outcome is complex and multifactorial and may reflect diversities in microbial pathogenicity along with the duration and severity of the fetal inflammatory response to microbial infection.

Chronic intra-amniotic infection with *U. parvum* serovars 3 and 6 in pregnant sheep induced chorioamnionitis and increased markers of systemic inflammation in the fetus; however, expression of pro-inflammatory genes and histological indices of systemic inflammation were higher in the chrorioamnion, amniotic fluid and lung tissue of fetuses exposed to serovar 6.^15,16^ In contrast, chronic exposure to intra-amniotic *U. parvum* serovar 3 (for 42 days) increased cerebral markers of DNA methylation but did not cause white matter inflammation or injury in fetal sheep. Similarly, in rhesus macaques exposed to intra-amniotic *U. parvum* (serovar 1) for 15–27 days, serial MRIs during the first 6 months after birth showed no significant differences in markers of regional brain growth or brain maturation.^17^

Collectively, these observations support the hypothesis that the pathogenesis of fetal infection/inflammation-induced brain injury in response to *U. parvum* is serovar dependant. This study aimed to determine whether histological markers of inflammation and injury differ in fetal sheep exposed to *U. parvum* serovars 3 and 6 from mid gestation until term equivalent age in relation to human brain development.

## Materials and methods

### Ethics statement

Experimental procedures were approved by the Animal Ethics Committees of University of Western Australia (UWA), Queensland University of Technology (QUT) and Cincinnati Children’s Hospital. The tissues used for this study were from the same animals used for Knox *et al*.^16^ The experiments are reported in accordance with the ARRIVE guidelines for reporting animal research.^18^

### *Ureaplasma* preparation and administration

*Ureaplasma parvum* serovars 3 and 6 used in this study were isolated from semen samples collected from consenting patients at Brisbane Wesley IVF Service. The serovars were confirmed from isolated samples using PCR assays.^16^ Aliquots of Ureaplasma positive semen samples were serially diluted in 10B broth, and after incubation, samples underwent serial dilutions during the exponential growth phase and were pooled then inoculated into 10B broth (20–30 mL of sample in 1 L of 10B broth). Samples underwent further culture until an alkaline colour shift was observed, then were pelleted by centrifugation at 4°C for 1 h (3700 g; Beckman J2-21 M/E), resuspended and washed in 2 mL cold sterile phosphate-buffered saline (PBS), and centrifuged again at 4°C for 15 min (Beckman Microfuge M). Resulting pellets were resuspended in 10B broth and pooled. The sample concentrations were determined in colony-forming unit (CFU/mL) using the serial dilution and drop plate method. Aliquots containing known CFU/mL were stored at −80°C prior to intra-amniotic injection. Before injection, the *U. parvum* was thawed and diluted in sterile cold PBS to concentrations of 2 × 10^4^ CFU (low dose) or 2 × 10^7^ CFU (high dose) in injection volumes of 2 mL. The inoculates were mixed, kept on ice and warmed immediately before intra-amniotic injection. Concentrated 10B broth was aliquoted and stored under the same conditions as the *U. parvum* samples for control injection.

### Experimental protocol

Merino ewes with singleton pregnancies were randomly allocated, using an online number generator, to one of five treatment groups at 55 days of gestational age to receive an ultrasound-guided intra-amniotic injection of either *U. parvum* serovar 3 low dose (S3LD; *n* = 5), *U. parvum* serovar 3 high dose (S3HD; *n* = 6), *U. parvum* serovar 6 low dose (S6LD; *n* = 8), *U. parvum* serovar 6 high dose (S6HD; *n* = 8), or control (concentrated 10B broth; *n* = 6). High and low dose concentrations for each serovar were included in the study to determine whether the degree of *U. parvum* colonization at delivery, as well as the severity of fetal inflammation and neural injury, is modifiable based on the initial bacterial load. *Ureaplasma parvum* injection at this timepoint correlates with the second trimester in sheep. This timing of exposure was chosen to correspond with human cohort studies that have identified *U. parvum* in amniotic fluid as early as the second trimester of pregnancy.^19^ All ultrasound imaging and intra-amniotic injections were performed at an agricultural facility where ewes had minimal human contact until they were transported to paddocks at a research facility at 90 days of gestation for delivery of fetuses and tissue collection at 125 days of gestation.

### Preterm delivery, tissue collection and Ureaplasma detection

At 125 days of gestation (term 145 days), ewes were anaesthetized using intravenous ketamine (12 mg/kg) and metatomidine (0.12 mg/kg), followed by spinal anaesthesia with 60 mg lignocaine. Delivery at this timepoint was chosen as it enabled investigation of the long-term effects of *U. parvum* infection on fetal growth and development, including its effects on the fetal brain. Assessment of long-term brain histopathology, beyond the first hours to days after an infectious or inflammatory insult, reflects a major gap in preclinical/animal studies of perinatal infection/inflammation.^20^ This study helps to address this important gap in knowledge. Investigators involved in the deliveries and post-mortem tissue collection were blinded to the treatment groups. Upon delivery, the foetus received a lethal dose of pentobarbitone (100 mg/kg) into the umbilical vein. Umbilical arterial blood was sampled for automated measurement of total and differential white blood cell (WBC) counts with correction for nucleated red blood cells. Amniotic fluid and fetal cerebrospinal fluid were aspirated through the fetal membranes and cisternae magna, respectively, using a sterile needle at the time of preterm delivery and frozen in liquid nitrogen, then stored at −80°C. The fetal brain was perfusion fixed *in situ* using 10% phosphate-buffered formalin before it was removed from the skull, weighed and immersion fixed in 10% phosphate-buffered formalin for a further 3 days and processed for histological assessment. The rate of fetal loss before the end of the experimental period was 15% and did not differ between groups. In cases of fetal loss, the individual was excluded from the study.

Samples of amniotic and cerebrospinal fluid were processed for Ureaplasma detection using established protocols described in Moss *et al*.^15^ and Knox *et al*.^16^ In brief, samples of amniotic and cerebrospinal fluid were thawed and serially diluted 10 times in 10B broth in 10-fold dilutions to determine the number of colony-forming units of Ureaplasma per millilitre of fluid. Ureaplasmas were quantified from 30 μL aliquots of the 10^−3^, 10^−4^ and 10^−5^ inoculated broth dilutions that were subcultured onto A8 agar. The plates were incubated in CO_2_ at 37°C for 48–72 h. Colonies were counted at ×40 magnification using a stereomicroscope (Leica Microsystems, North Ryde, NSW, Australia).

### Immunohistochemistry analysis

Using a brain mould, the whole brain (left and right hemispheres) was divided into four coronal portions before undergoing processing and embedding using a standard paraffin tissue preparation. Blocks from the forebrain, ∼23 mm anterior to stereotaxic zero, with a clearly visible striatum, were sectioned into 8-µm thick sections using a microtome (Leica Microsystems, VIC, Australia). The slides were dewaxed in xylene, rehydrated in increasing concentrations of ethanol and washed in 0.1 mol/L PBS. Antigen retrieval was performed in sodium citrate buffer (10 mM sodium citrate, pH 6.0). Endogenous peroxidase quenching and blockade of non-specific antigen binding on sections were performed using 0.1% hydrogen peroxide in methanol and 3% normal goat serum, respectively.

Sections were labelled with 1:200 rabbit anti-glial fibrillary acidic protein (GFAP, Cat#: Ab68428, Abcam, Cambridge, Australia), 1:200 rabbit anti-ionized calcium binding adaptor molecule 1 (Iba-1, cat#: Ab178846, Abcam), 1:200 rabbit anti-oligodendrocyte transcription factor-2 (Olig-2, cat#: Ab109186, Abcam), 1:200 mouse anti-cyclic nucleotide 3′ phosphodiesterase (CNPase, cat#: Ab6319, Abcam), 1:200 mouse anti-adenomatous polyposis coli clone (CC1, cat#: OP90-100UG, MerkMillipore, Burlington, MA, USA), 1:200 rat anti-myelin basic protein (MBP, cat#: MAB395, MerkMillipore) and 1:200 rabbit anti-neuronal nuclei (NeuN, cat#: Ab177487, Abcam). Sections were incubated overnight at 4°C. Thereafter, sections were incubated in biotin-conjugated goat anti-rabbit IgG (cat#: 111-065-144, Jackson ImmunoResearch Laboratories Inc., PA, USA), goat anti-rat IgG (cat#: 112-035-143, Jackson ImmunoResearch Laboratories Inc.) or goat anti-mouse IgG (Vector Laboratories, Inc, CA, USA) for 3 h at room temperature before being incubated in avidin-biotin complex (Vector Laboratories Inc; CA, USA) for 45 min at room temperature. Sections were reacted with 3,3′-diaminobenzidine tetrahydrochloride (DAB, MP Biomedicals LLC; OH, USA). The reaction was stopped in PBS before the slides were dehydrated in xylene and increasing concentrations of ethanol, mounted in dibutyl phthalate polystyrene xylene and cover-slipped. All staining was performed on fresh cut sections. Staining protocols for all antibodies were optimized in pilot trials before performing large batch immunohistochemical staining on all subjects.

Astrocytes (GFAP+ cells), microglia (Iba-1+ cells), oligodendrocytes (Olig2+, CNPase+ and CC1+ cells), myelin density (MBP), neurons (NeuN+ cells) and cell death (TUNEL+ cells) were visualized and imaged at 40× magnification using light microscopy (Olympus BX41; Camera: Olympus DP25, Olympus, Tokyo, Japan) and cellSens Standard (Version 2.3, Olympus).

**Figure 1:**
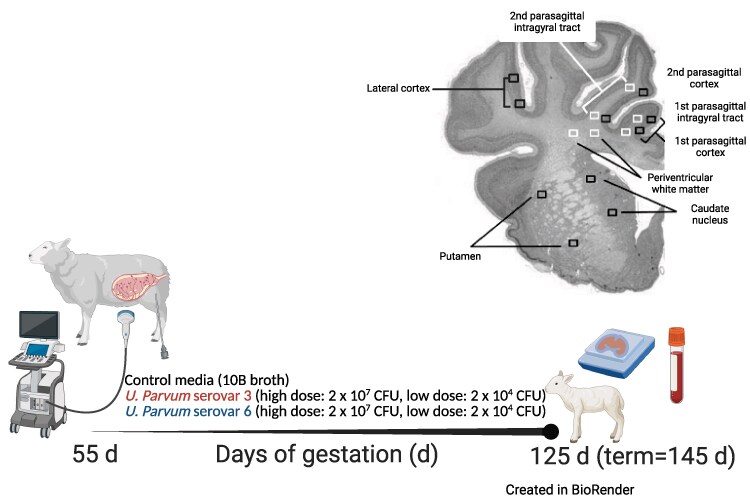
**Experimental design and brain regions of interest (ROIs) for histological analysis.** At 55 days (0.4) of gestation, pregnant ewes were randomly assigned to receive an ultrasound-guided intra-amniotic injection of either control media (10B broth; *n* = 6), *Ureaplasma parvum* serovar 3 [high dose: 2 × 10^7^ CFU (*n* = 5), low dose: 2 × 10^4^ CFU (*n* = 6)] or *U. parvum* serovar 6 [high dose: 2 × 10^7^ CFU (*n* = 8), low dose: 2 × 10^4^ CFU (*n* = 8)]. At 125 days (0.8) of gestation, umbilical arterial blood and fetal brains were collected and processed for analysis. Brain ROIs were sampled from histological sections taken 23 mm anterior to stereotaxic zero. White boxes represent the white matter ROIs that were captured within the periventricular and first and second intragyral white matter tracts. Black boxes represent grey matter ROIs that were captured within the caudate nucleus, putamen and premotor cortex. Created in BioRender. Galinsky, R. (2025) https://BioRender.com/p80o123.

For white matter regions of interest (ROIs) (Olig-2, CNPase, CC1, MBP, Iba1 and GFAP staining), images were taken in the periventricular white matter and parasagittal first and second intragyral white matter tracts (Fig. 1). For grey matter ROIs (NeuN, Iba1 and GFAP staining), images were taken in the first and second parasagittal gyri, the lateral gyrus and within the caudate nucleus and putamen of the striatum (Fig. 1). Images were imported into ImageJ software for quantifying area fraction of staining (MBP density) and manual cell quantification (Iba1, Olig2, NeuN, GFAP and CC1). Microglia (Iba-1 positive cells) showing ramified and amoeboid morphology were included in our assessment of microglia per field of interest. Iba-1 positive microglia displaying an activated phenotype were characterized by an enlarged amoeboid shaped cell body with ≤1 branching process.^21,22^ Only GFAP positive astrocytes containing a positively stained nucleus were counted. The area fraction of MBP positive staining was determined by converting original files into 8-bit images then using a standard intensity threshold to calculate the percentage area fraction of MBP staining. Coherence analysis of MBP-stained images to measure myelin anisotropy, were conducted using the OrientationJ plugin, as previously described.^23^ Orientation characterizes the isotropic/anisotropic properties of a ROI in an image based on the evaluation of the structure tensor. The OrientationJ measure tool was used to select the areas in Fiji to define the region through which OrientationJ creates the best fitting ellipse that represents the image gradient. The rectangular selection tool was used to measure coherence within the whole periventricular and intragyral white matter section (global MBP anisotropy).

ApopTag was used to detect single and double-stranded breaks in DNA associated with apoptosis.^24^ Staining was carried out according to manufacturer’s instructions (MilliporeSigma’s 7100, ApopTag Peroxidase in Situ Apoptosis Detection Kit). In brief, tissue was dewaxed in xylene, rehydrated in increasing concentrations of ethanol and washed in 1× PBS. The tissue was then pre-treated with proteinase K for 15 min, washed in PBS and background peroxidase activity quenched in 3.0% hydrogen peroxide for 5 min. The equilibration buffer was added for 10 min, before the TdT enzyme was added and left for 1 h at 37°C. The reaction was stopped in stop buffer for 10 min, then washed before adding the anti-digoxigenin conjugate for 30 min at room temperature. Finally, peroxidase substrate was added for 4 min before the tissue was counterstained in 50% haematoxylin (cat#: MH1NPR, Trajan Scientific, VIC, Australia), dehydrated in xylene followed by increasing concentrations of ethanol, mounted in dibutyl phthalate polystyrene xylene and cover-slipped. Total numbers of terminal deoxynucleotidyl transferase-mediated dUTP nick end labelling (TUNEL+) cells in each ROI were counted. The areas that were sampled for quantifying total numbers of TUNEL positive cells are highlighted in Supplementary Fig. 1. The total area sampled did not differ between subjects within or between the groups. For each cortical gyri, TUNEL positive cells were counted within layers 3–5, starting at the base and ending at mid-point of each gyrus. For the caudate nucleus and putamen, total TUNEL positive cells were counted within the areas that bordered the internal and external capsules. For the periventricular white matter, total TUNEL positive cells were quantified within the area that was bordered by the first, second and third parasagittal sulci, and the subventricular zone (Supplementary Fig. 1).

### Immunofluorescence of microglial and astrocyte activation

Slides were baked at 60°C for 1 h then dewaxed in xylene, rehydrated in increasing concentrations of ethanol and washed in 1× PBS. Antigen retrieval was performed in citrate buffer (pH 6) using a microwave for 15 min. Non-specific antigen blocking was performed using 10% normal goat serum. Sections were labelled with 1:200 mouse anti Iba1 (cat#: ab 283319), or 1:200 mouse anti-GFAP (cat#: Ab224659, Abcam) overnight at 4°C. Sections were incubated with an Alexa Fluor-488-conjugated secondary antibody (1:200, cat#: 115-545-003, Jackson ImmunoResearch Laboratories Inc.) for 2 h at room temperature then labelled with 1:200 rabbit anti-phosphorylated signal transducer and activator of transcription 3 (STAT3 cat#: 9145, Cell Signalling, MA, USA) overnight at 4°C to identify pro-inflammatory M1 microglia and A1 astrocytes. Sections were incubated with an Alexa Fluor-594-conjugated secondary antibody (1:200, cat#: 111-585-003, JacksonResearch Laboratories Inc.) for 2 h at room temperature then washed in 1× PBS 3× for 10 min before being incubated with the nuclear stain HOECHST (1:1000 diluted in 1× PBS; Invitrogen, USA) for 5 min, then washed for 5 min in 1× PBS. Sections were cover-slipped using DAKO anti-fade fluorescent mounting medium (Agilent Technologies, Australia) and allowed to dry. Negative controls that did not contain the target antibody were included to confirm the absence of non-specific staining (Supplementary Fig. 2).

Sections with fluorescently double-labelled microglia (IBA+/p-STAT3+) and astrocytes (GFAP+/p-STAT3+) were visualized and imaged at 40× magnification using a fluorescence microscope (Olympus BX41; Camera: Olympus DP25; Burner: U-LGPS, Olympus, Tokyo, Japan) and cellSens Standard imaging software (Version 2.3, Olympus). Astrocytes that were positive for both GFAP and STAT3 were quantified for each white and grey matter ROI (Fig. 1) from two sections per subject. All imaging and cell counts were performed by an assessor who was blinded to the treatment group through coding of slides/files by an independent study manager.

### Statistical analysis

There were no differences in markers of systemic inflammation or brain histological outcomes between the low and high dose *U. parvum* groups for either serovar. Therefore, data from the high- and low-dose groups were combined. Statistical analysis was undertaken using GraphPad Prism (Version 9.5.1, GraphPad Software, CA, USA). Data were tested for normality using the Shapiro–Wilk test. For non-parametric data, between group comparisons were performed using Kruskal–Wallis tests. For neuropathological data, between group comparisons were performed using a two-way ANOVA with treatment and brain region as individual factors. The Benjamini–Hochberg correction or Tukey’s test was used for *post hoc* comparisons. Power calculation for mature myelinating (CC1+) oligodendrocytes showed 85% power to detect a minimum difference of 30 cells per field with an α of 0.05. Data are presented as mean ± SEM. Statistical significance was accepted when *P* < 0.05.

## Results

### Post-mortem data and white blood cell counts at delivery

**Figure 2:**
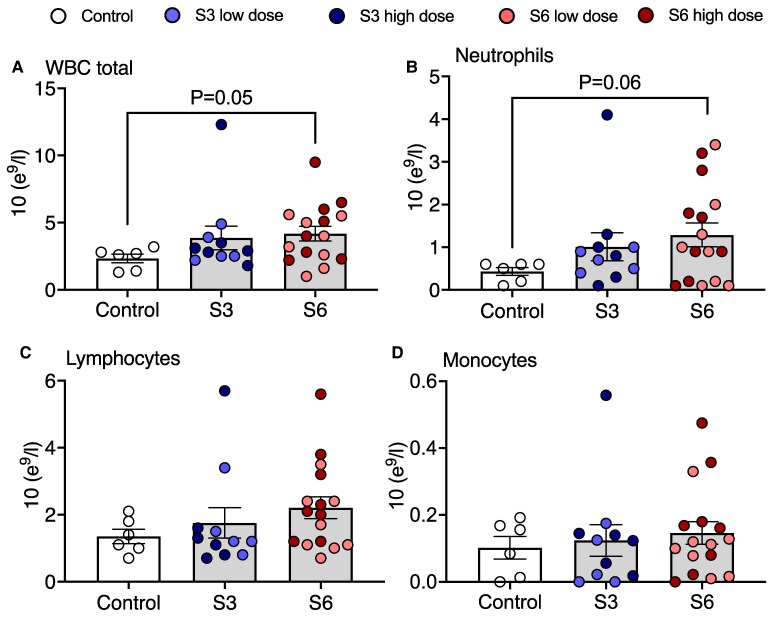
**White blood cell analysis in *Ureaplasma parvum* serovar 3 and serovar 6-exposed subjects.** Total white blood cells (**A**), neutrophils (**B**), lymphocytes (**C**) and monocytes (**D**) were compared between control (white circles, *n* = 6), serovar 3 [low dose, light blue circles (*n* = 5); high-dose, dark blue circles (*n* = 6)] and serovar 6 [low-dose, light red circles (*n* = 8); high dose, dark red circles, (*n* = 8)]-exposed subjects. Data are individual subject means ± SEM. Statistical analysis: Kruskal–Wallis test with Benjamini–Hochberg procedure.

Ureaplasmas were detected in the amniotic fluid of all animals injected intra-amniotically with *U. parvum* serovars 3 and 6 (Table 1). There were no differences in *U. parvum* CFU within the amniotic fluid between the low and high dose *U. parvum* groups for either serovar (Table 1). There were no differences in brain weight, body weight or sex ratios between the groups (Table 1). Analysis of cerebrospinal fluid found *U. parvum* in two subjects, one from the serovar 3 cohort (1.10E^+05^) and one from the serovar 6 cohort (1.30E^+05^). Analysis of maternal blood showed no trace of *U. parvum*. Total circulating white blood cell counts were not significantly higher in the *U. parvum* serovar 6 group compared to control (*P* = 0.05, Fig. 2). Numbers of circulating neutrophils were not significantly higher in the *U. parvum* serovar 6 group compared to control (*P* = 0.06, Fig. 2). There were no differences between groups for numbers of circulating lymphocytes and monocytes (Fig. 2).

**Table 1:** Group demographics, including sex ratios, fetal body and brain weights (means ± SE), amniotic fluid *Ureaplasma parvum* colonization (means and range) and incidence of CSF *U. parvum* detection

	Control	Serovar 3 (*n* = 11)		Serovar 6 (*n* = 16)	
Dose		High	Low	High	Low
Numbers (*n*)	6	6	5	8	8
Sex ratio (M:F)	4:2	2:4	3:2	4:4	4:4
Body weight (kg)	2.86 ± 0.12	2.56 ± 0.13	2.58 ± 0.09	2.71 ± 0.10	2.65 ± 0.12
Brain weight (g)	43.9 ± 1.6	43.0 ± 1.5	45.3 ± 0.2	45.2 ± 1.6	46.3 ± 1.4
Amniotic fluid *U. parvum* colonization (CFU/mL)	Not detected	1.39 × 10^6^ (1.4 × 10^5^–4.5 × 10^6^)	3.75 × 10^6^ (1.5 × 10^5^–2.6 × 10^5^)	1.39E^+06^ (2.0 × 10^5^–3.6 × 10^6^)	2.78 × 10^5^ (1.3 × 10^5^–5.2 × 10^5^)
*U. parvum* detected in CSF (incidence)	0/6	1/6	0/5	1/8	0/8

### White matter pathology

#### Microglia and astrocytes

**Figure 3:**
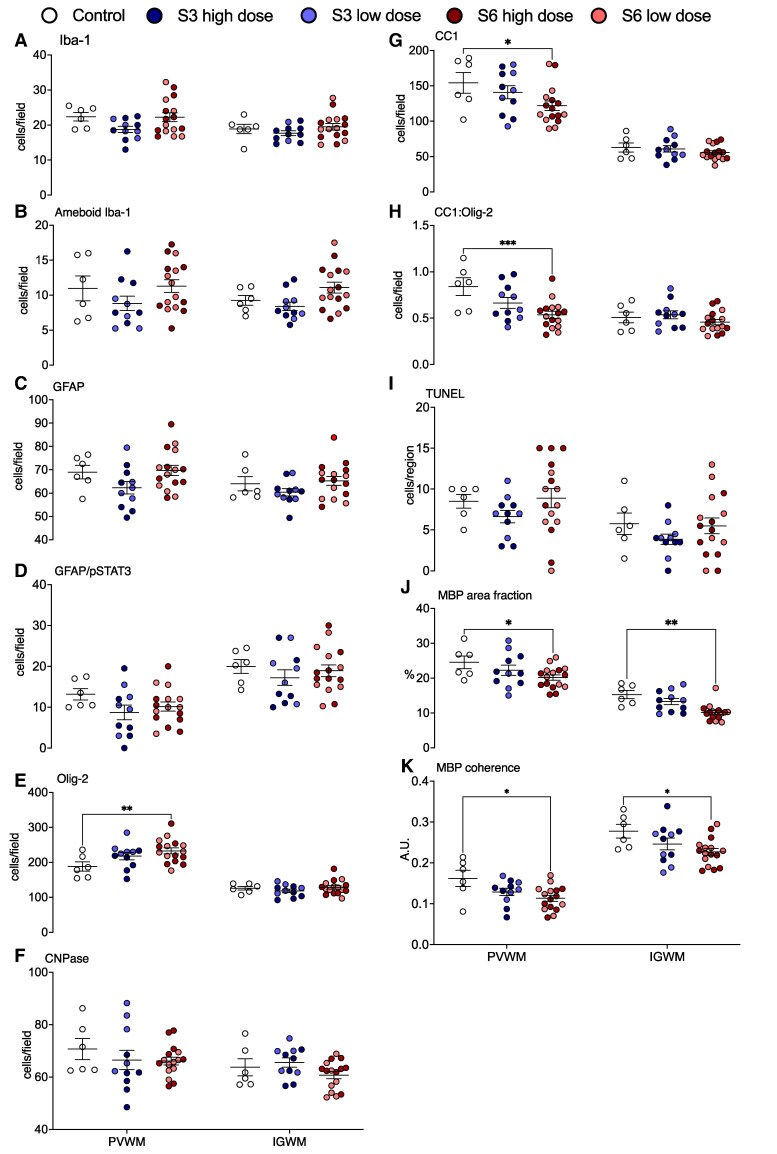
**White matter histopathology.** Numbers of total (**A**), amoeboid (**B**) Iba-1+ microglia, GFAP+ (**C**) and GFAP/phosphorylated signal transducer and activator of transcription (pSTAT)3+ astrocytes (**D**), Olig-2+ (total; **E**), CNPase (immature and mature; **F**) and CC1+ (mature; **G**) oligodendrocytes, proportions of mature (CC1:Olig2+; **H**) oligodendrocytes, TUNEL+ (apoptotic; **I**) cells, MBP % area fraction (density; **J**) and coherence (anisotropy; **K**) in the periventricular (PVWM) and intragyral white matter tracts (IGWM) in control (white circles, *n* = 6), *Ureaplasma parvum* serovar 3 [low dose (*n* = 5), light blue circles; high-dose (*n* = 6), dark blue circles] and serovar 6 [low-dose (*n* = 8), light red circles; high dose (*n* = 8), dark red circles]-exposed subjects. Data are individual subject means ± SEM. Statistical analysis: two-way ANOVA with Tukey’s multiple comparison test. *P < 0.05, **P < 0.01, ***P < 0.001.

**Figure 4:**
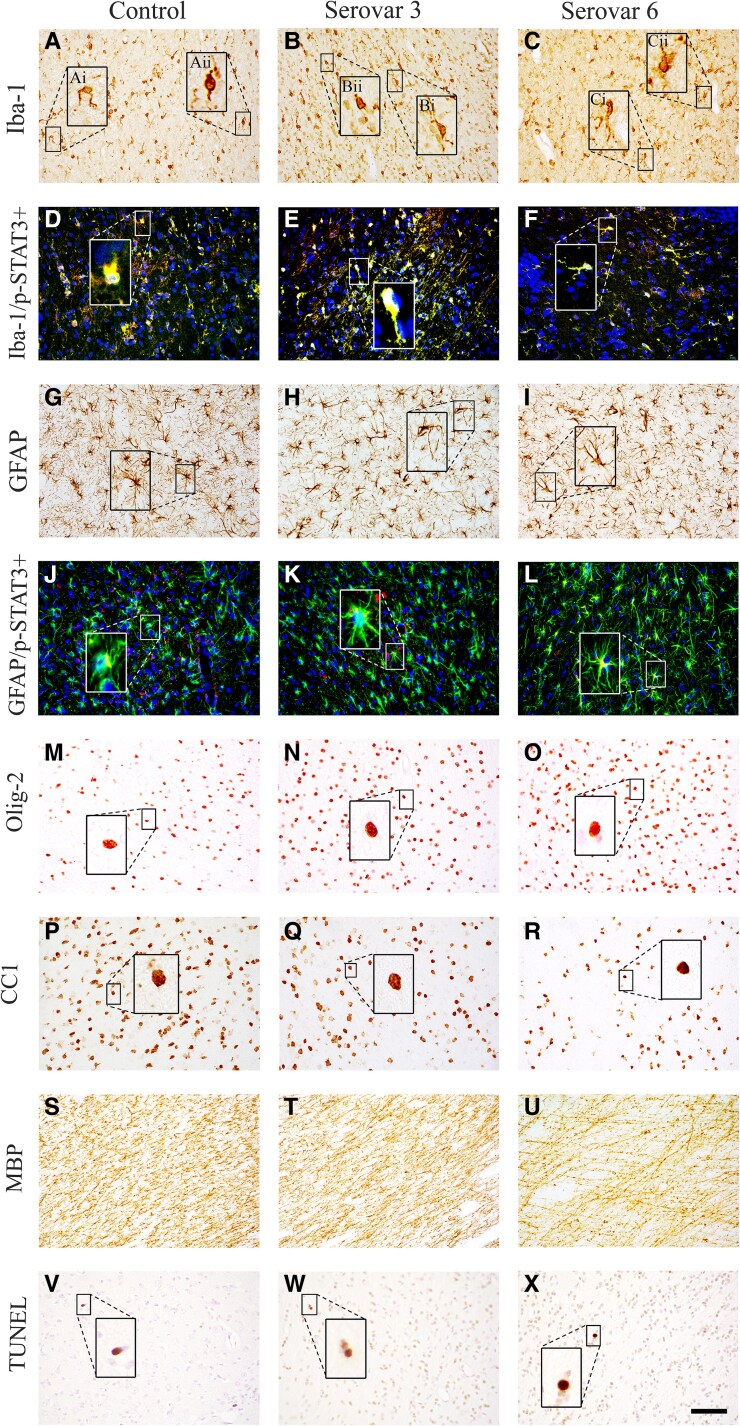
**Photomicrographs of white matter immunohistochemistry.** Representative photomicrographs showing Iba-1 (**A–C**), Iba-1/phosphorylated (p)-STAT3 [activated microglia = yellow (**D–F**); green = Iba-1, red = p-STAT3, blue = 4′,6-diamidino-2-phenylindole (DAPI)], GFAP (**G–I**), GFAP/p-STAT3 (activated astrocytes = yellow, **J–L**; green = GFAP, red = p-STAT3, blue = DAPI), Olig-2+ (**M–O**), CC1 (**P–R**) and MBP (**S–U**) and TUNEL (**V–X**) staining in the periventricular white matter (PVWM) in control, *Ureaplasma parvum* serovar 3 and serovar 6-exposed subjects. Representative images of ramified (**Ai**, **Bi**, **Ci**) and amoeboid (**Aii**, **Bii**, **Cii**) microglia shown in high magnification. Scale bar is 50 μm.

Numbers of microglia (Iba-1+ cells) and GFAP positive astrocytes within the intragyral and periventricular white matter tracts were not different between groups (Figs 3 and 4). Similarly, there were no differences in numbers of amoeboid microglia, phosphoSTAT3+/Iba-1+ microglia (Iba-1+/p-STAT3+) or phosphoSTAT3+/GFAP+ astrocytes (GFAP+/p-STAT3+) between groups (Figs 3 and 4).

#### Oligodendrocytes and myelin density

In the serovar 6 group, total numbers of Olig-2+ oligodendrocytes in the periventricular white matter were higher than control (*P* < 0.01, Figs 3 and 4). In the serovar 3 group, total numbers of Olig-2+ oligodendrocytes were not different from control (Figs 3 and 4). There were no differences in total numbers of Olig-2+ oligodendrocytes in the intragyral white matter between groups. Numbers of immature and mature CNPase+ oligodendrocytes in the intragyral and periventricular white matter tracts were not different between groups (Figs 3 and 4). Numbers of mature CC1+ oligodendrocytes in the periventricular white matter were lower in the serovar 6 group compared to control (*P* < 0.05, Figs 3 and 4), but were not significantly reduced in the intragyral white matter. In the serovar 3 group, numbers of mature CC1+ oligodendrocytes did not differ from control in the intragyral and periventricular white matter tracts. The proportion of mature oligodendrocytes (CC1: Olig-2+ cells) within the periventricular white matter was lower in the serovar 6 group compared to control (*P* < 0.001) but was not significantly lower in the intragyral white matter (Figs 3 and 4). The proportion of mature oligodendrocytes within the periventricular and intragyral white matter tracts was not different between the serovar 3 and control groups. MBP density and anisotropy in the periventricular and intragyral white matter tracts were lower in the serovar 6 group compared to control (*P* < 0.05, Figs 3 and 4). MBP density and anisotropy did not differ between the serovar 3 group compared to control (Figs 3 and 4).

### Premotor cortex and striatum

#### Microglia and astrocytes

**Figure 5:**
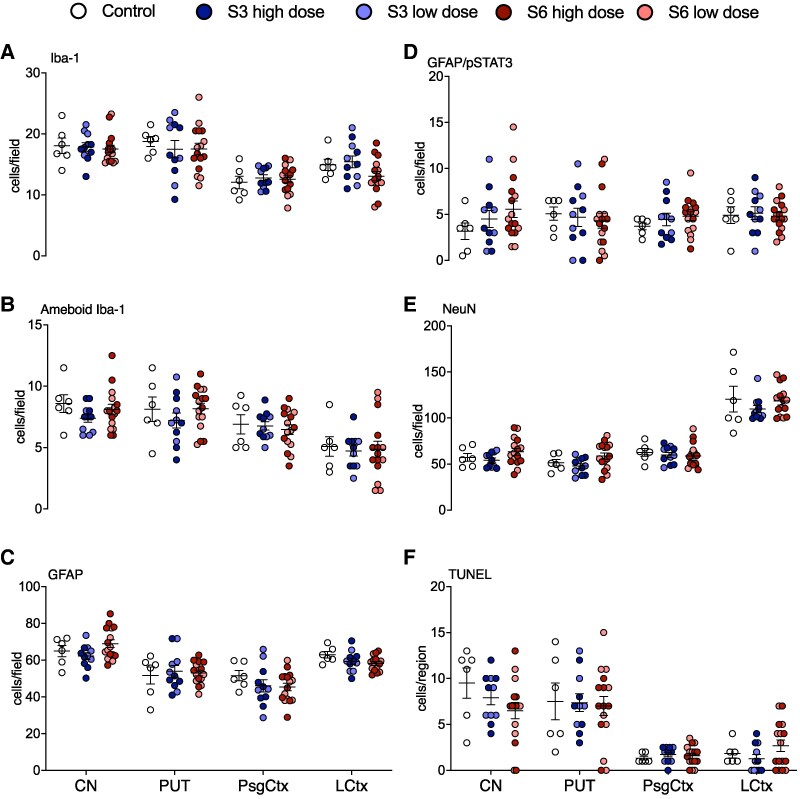
**Grey matter histopathology.** Numbers of total (**A**) and amoeboid (**B**) Iba-1+ microglia, GFAP+ (**C**) and GFAP/phosphorylated (p)STAT3+ (**D**) astrocytes, NeuN+ neurons (**E**) and TUNEL+ (apoptotic; **F**) cells in the caudate nucleus (CN), putamen (PUT), parasagittal (PsgCtx) and lateral (LCtx) cortices in control (white circles, *n* = 6), *Ureaplasma parvum* serovar 3 [low dose (*n* = 5), light blue circles; high-dose (*n* = 6), dark blue circles] and serovar 6 [low-dose (*n* = 8), light red circles; high dose (*n* = 8), dark red circles]-exposed subjects. Data are individual subject means ± SEM. Statistical analysis: two-way ANOVA with Tukey’s multiple comparison test.

**Figure 6:**
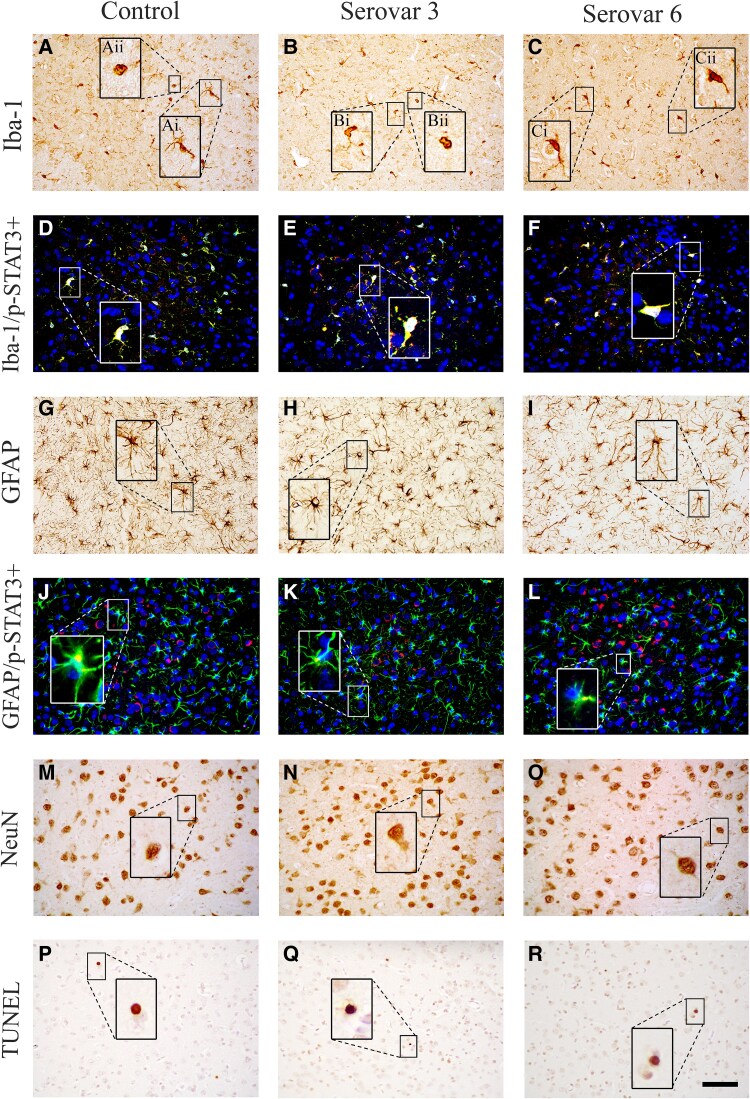
**Photomicrographs of grey matter immunohistochemistry.** Representative photomicrographs showing Iba-1 (**A–C**), Iba-1/phosphorylated (p)-STAT3 [activated microglia = yellow, **D–F**; green = Iba-1, red = phosphoSTAT3, blue = 4′,6-diamidino-2-phenylindole (DAPI)], GFAP (**G–I**), GFAP/phosphoSTAT3 (activated astrocytes = yellow, **J–L**; green = GFAP, red = p-STAT3, blue = DAPI), NeuN (**M–O**) and TUNEL (**P–R**) staining in the premotor cortex. Representative images of ramified (**Ai**, **Bi**, **Ci**) and amoeboid (**Aii**, **Bii**, **Cii**) microglia shown in high magnification. Areas of analysis include: caudate nucleus (CN), putamen (PUT), parasagittal (PsgCtx) and lateral (LCtx) cortices. Scale bar is 50 μm.

Numbers of Iba-1+ microglia and GFAP+ astrocytes within the caudate nucleus and putamen of the striatum and premotor cortex were not different between groups (Figs 5 and 6). Similarly, there were no differences in numbers of amoeboid microglia or phosphoSTAT3+/GFAP+ astrocytes between groups. Qualitative assessment of phosphoSTAT3+/Iba-1+ microglia suggested that there were no differences in microglial activation between groups within the periventricular white matter (Fig. 6D–F).

#### Neuronal numbers

Numbers of neurons within the caudate nucleus and putamen of the striatum and the premotor cortex were not different between groups (Figs 5 and 6).

#### Cell death

There were no differences in total numbers of TUNEL+ apoptotic cells between groups within the white and grey matter ROIs (Figs 3 and 5). Overall, there were no areas of white or grey matter within the selected ROIs that had overt signs of infarct, pyknosis, karyorrhexis, vacuolation or cellular oedema.

## Discussion

Chronic intra-amniotic infection with *U. parvum* serovar 6, but not serovar 3, from 0.4 of gestation, caused a reduction in the numbers of mature myelinating oligodendrocytes, and reduced myelin density and myelin integrity in the large white matter tracts of late gestation fetal sheep. *Ureaplasma parvum* is among the most common bacterial species isolated from the amniotic fluid in pregnancies complicated by infection.^8,9^ However, evidence linking *U. parvum* infection and brain injury and/or adverse neurodevelopment is unclear.^3,14^ This most likely reflects the complex and multifactorial nature of microbial infection, which may include the duration of pathogen exposure, severity of the pathogen and heterogeneity of antenatal infections that are likely to have variable effects on the fetal inflammatory response.^20^ Here, we have shown that exposure to *U. parvum* serovars 3 and 6, two of the most common Ureaplasma serovars detected in the human female reproductive tract, had differential effects on markers of white matter development in the fetal brain.

Despite observing reduced numbers of mature (CC1+) oligodendrocytes in serovar 6-exposed fetuses, numbers of total (Olig-2+) oligodendrocytes were increased in serovar 6-exposed fetuses compared to controls, which likely reflects restorative proliferation of oligodendrocytes. These data are consistent with previous studies in preterm humans and other species, which showed that exposure to inflammation promotes acute cell loss followed by restorative proliferation of oligodendrocytes that subsequently fail to mature into myelinating cells.^25-28^

Consistent with the reduction in numbers of mature oligodendrocytes in serovar 6-exposed fetuses, the observed reduction in myelin density and myelin anisotropy indicates a reduction in myelin fibre integrity. These data are consistent with observations in human MRI studies that associated exposure to antenatal inflammation with reductions in white matter development and white matter integrity.^29-31^ Indeed, oligodendrocyte maturation is vital to axonal development and function.^32^ The combined loss of mature myelinating oligodendrocytes, reduced myelin density and reduced myelin anisotropy observed in serovar 6-exposed fetuses could ultimately result in reduced functional integrity of neuronal axons and impaired neuronal growth, development and function. Interestingly, we observed a reduction in myelin density in the intragyral white matter in serovar 6-exposed fetuses compared to controls but did not observe a significant reduction in numbers of mature/myelinating CC1 positive oligodendrocytes. These data raise the possibility that mature myelination oligodendrocytes in serovar 6-exposed fetuses had a reduced capacity to produce myelin. Similar observations were made in fetal sheep exposed to hypoxia–ischaemia^25^ and in a rodent model of demyelinating injury.^33^ We did not observe loss of neuronal nuclei in serovar 3 or 6-exposed fetuses compared to controls, indicating that there was no overt neuronal loss in fetuses exposed to *U. parvum*. However, analyses of neuronal numbers were limited to the somatosensory cortex and striatum, areas that have been shown to be susceptible to inflammation-induced pathology in human and animal studies.^25,29,34,35^ Assessment of other areas of grey matter that are susceptible to injury, particularly deep grey matter nuclei such as the thalamus and hippocampus, in addition to evaluating axonal development, neuronal growth, arborization and function should also be considered in future studies.

Cerebrospinal fluid analysis found *U. parvum* in only two CSF samples collected 70 days after intra-amniotic injection. These data are consistent with CSF analyses in previous studies in fetal sheep, rhesus macaques, human neonates and brain tissue samples from non-human primate studies that have all reported limited transfer of *U. parvum* into the CSF and brain tissue.^36-39^ Indeed, previous studies in fetal sheep showed that accumulation of *U. parvum* was mainly observed in the placenta, umbilical cord, fetal gut and lungs.^15,16^ Together, these data suggest that there is limited transfer of *U. parvum* across the blood–brain barrier and that its effects on brain development may instead be mediated through peripheral immune activation. Indeed, studies in humans and animals have shown that exposure to *U. parvum* during the perinatal period is associated with increased levels of circulating inflammatory markers (e.g. increased circulating white blood cells and cytokines), along with increased markers inflammation in peripheral organs, such as the lungs, gut and placenta.^16,36,40-42^ We did not measure circulating cytokines in this study, but we did observe trends for increased numbers of neutrophils (*P* = 0.06) and total white blood cells (*P* = 0.05) in serovar 6-exposed fetuses compared to controls. Indeed, systemic inflammation can trigger brain inflammation via several mechanisms. These include blood–brain barrier disruption, resulting in increased permeability to infiltrating immune cells; entry via circumventricular organs, such as the choroid plexus, which lacks a blood–brain barrier; and via transporters responsible for trafficking of specific cytokines between the circulation and brain.^43,44^ Once inside the brain, inflammatory molecules (e.g. cytokines) are capable of binding to receptors on neurons and glial cells to trigger a local inflammatory response that causes cell death and/or impairs cellular maturation.^45^ At the time of tissue collection, there were no differences in numbers of total or phophoSTAT3 positive (A1-reactive) astrocytes, amoeboid (activated) microglia or apoptotic cells in the *U. parvum*-exposed fetuses compared to controls. These data suggest that 70 days after intra-amniotic Ureaplasma injection, histological signatures of central nervous system inflammation and injury may have resolved. Moreover, in future studies, temporal assessments of systemic and CSF cytokines and white blood cell counts throughout the experimental period, including assessment of the proportion of immature and total neutrophils, would provide important insight into the temporal profile of the systemic and central nervous system immune responses to *U. parvum* infection.

Exposure to *U. parvum* serovar 3 had limited effects on histological markers of white matter development relative to serovar 6. To the best of our knowledge, this is the first study comparing exposure to *U. parvum* serovars 3 and 6 on markers of brain inflammation and pathology. The lack of brain pathology after 70 days of exposure to serovar 3 in this study is consistent with previous studies that showed no effect of intra-amniotic exposure to serovar 3 on markers of systemic and brain inflammation, and cortical and white matter development in fetal sheep after 7^37^ or 42 days.^16^ Similarly in pregnant rhesus macaques, intra-amniotic inoculation (for 3–7 or 15–27 days of gestation) with *U. parvum* serovar 1, another serovar found in the female urogenital tract, had limited effects on fetal and postnatal brain growth and white matter development.^17,36^ In contrast, 14 day old mouse pups exposed to an intra-amniotic injection of *U. parvum* serovar 3, immediately before birth, displayed increased numbers of cortical microglia and loss of GABA-ergic interneurons. However, evidence of peripheral inflammation was also observed in the vehicle group, which raises the possibility that the pro-inflammatory and injurious effects were not exclusively mediated by Ureaplasma exposure.^46^ Importantly, we have previously shown no effects of vehicle (10B broth) exposure on markers of peripheral inflammation.^15^ Overall, these data raise the possibility that the pathogenic effects of Ureaplasma infection on the preterm brain could be serovar dependant. These data are supported by previous studies that investigated markers of systemic inflammation, chorioamnionitis and lung inflammation after chronic exposure (45 days) to *U. parvum* serovars 3 and 6 in fetal sheep. The severity of chorioamnionitis and lung inflammation was, on average, higher in fetuses exposed to serovar 6 compared to serovar 3.^15^ However, exposure to both serovars caused chorioamnionitis and lung inflammation compared to the vehicle group, but the effects of serovars 3 and 6 were not directly compared against each other and the doses of *U. parvum* were higher compared to the doses used in this study.^15^

The pathogenicity of *U. parvum* infection depends on the size variability of the multiple banded antigen (MBA) gene and protein.^16,47,48^ The MBA is a surface-exposed lipoprotein of *U. parvum* and the main virulence factor responsible for triggering an immune response after recognition by the host organism.^49^ Variation in the size of the MBA gene and protein has been postulated to enable ureaplasmas to evade the immune system and facilitates their survival *in vivo*.^50,51^ In pregnant sheep that underwent intra-amniotic injection with *U. parvum*, the number of MBA size variants was inversely related to the severity of fetal tissue inflammation.^16^ Collectively, this raises the possibility that reduced variability in the MBA of serovar 6-exposed fetuses compared to the serovar 3-exposed group contributed to the increase in white matter pathology observed in serovar 6-exposed fetuses. Consistent with this finding, previous studies in fetal sheep exposed to *U. parvum* serovar 6 showed increased indices of fetal infection/inflammation, including increased prevalence and severity of histological chorioamnionitis, funisitis and meconium-stained amniotic fluid compared to fetuses exposed to serovar 3.^16^ These observations highlight the potential role of MBA size variations in determining the severity of Ureaplasma infection and may contribute to explaining why some fetuses exposed to ureaplasmas have adverse neurodevelopmental outcomes while others do not.^14^ To the best of our knowledge, comparisons of MBA/mba variation between *U. parvum* serovars 3 and 6 are limited. Indeed, there are data that suggest that size variation between *U. parvum* serovars 3 and 6 does not differ and that variation in MBA/mba size, independent of the Ureaplasma serovar, was responsible the severity inflammation.^52^ However, these data were derived from a small sample of clinical isolates collected from the chorioamnion of pregnant females (*n* = 19).^47^ Moreover, variation in MBA size in isolates collected from the fetal lung, amniotic fluid and chorioamnion was shown to increase with the duration of inoculation during gestation.^53^ This supports our suggestion that the pro-inflammatory effects of *U. parvum* serovar 6 had likely resolved by the time blood and brain tissue were collected. Indeed, it is possible that an increase in MBA size variation after 70 days of amniotic fluid colonization could underpin the lack of an inflammatory or cell death signature in the brain at the time of tissue collection.

We observed no effect of *U. parvum* dose on either markers of systemic inflammation (e.g. total or differential white blood cells) or brain pathology (e.g. markers of white and grey matter pathology). However, our analysis was limited a single timepoint (70 days) after *U. parvum* exposure. Consistent with this, there were no differences *U. parvum* colonization in the amniotic fluid between fetuses exposed to an initial low or high dose of *U. parvum*. This observation is consistent with previous data that showed no effect of *U. parvum* dose on the incidence and severity of fetal inflammation, or the degree of colonization of the amniotic fluid, chorioamniotic membranes and the fetal lungs.^16^ Similar observations have been made in rodent models of reproductive tract infections, where the dose of *U. parvum* was not associated with infection severity.^54^ The lack of a simple dose response likely reflects the complexity and dynamic nature of interactions between live microorganisms and their host. Moreover, amniotic fluid has been shown to support Ureaplasma growth.^48^ Indeed, Ureaplasma growth kinetics show an initial log increase in the first weeks after amniotic colonization, followed by a plateau, and then a decline in colonization.^48^ Moreover, the prolonged duration of intra-amniotic inoculation to delivery could be another contributor to the similar degree of Ureaplasma colonization observed between the high and low dose regimes.

In conclusion, amniotic fluid colonization by *U. parvum* serovar 6, but not serovar 3, reduced numbers of mature myelinating oligodendrocytes, myelin density and myelin integrity in the preterm fetal sheep brain. These cellular changes reflect serovar dependent impairment of white matter development. Overall, these data demonstrate that greater emphasis needs to be placed on the taxonomy of Ureaplasma infection when designing and interpreting future clinical and preclinical studies of perinatal infection/inflammation.

## Supplementary Material

fcaf182_Supplementary_Data

## Data Availability

The datasets used during the current study are available from the corresponding author upon reasonable request.
