# Supplementary material for: Intra-amniotic infection with Ureaplasma parvum causes serovar-dependent white matter damage in preterm fetal sheep
Source: Brain Commun. 2025 May 24;7(3):fcaf182. doi: 10.1093/braincomms/fcaf182 (PMC12130620; doi:10.1093/braincomms/fcaf182)
Supplement: fcaf182_Supplementary_Data [file fcaf182_supplementary_data.docx]

Supplementary figure 1. Brain regions sampled from histological sections taken 23 mm anterior to stereotaxic zero for quantifying numbers of TUNEL+ cells/region. White areas represent the periventricular and first and second intragyral white matter tracts. Black areas represent the caudate nucleus, putamen and lateral cortex

Merged

STAT3

Iba-1

DAPI

Merged

STAT3

GFAP

DAPI


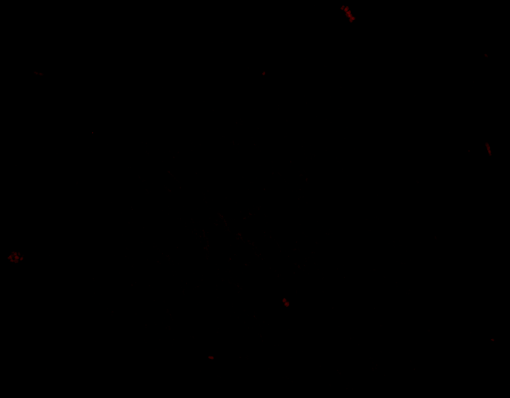

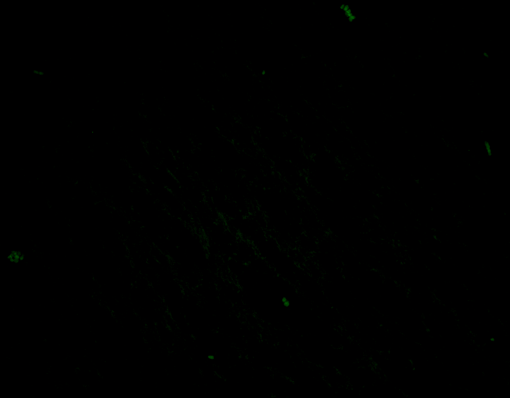

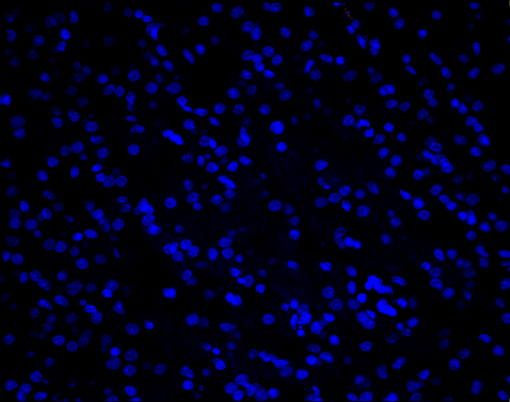

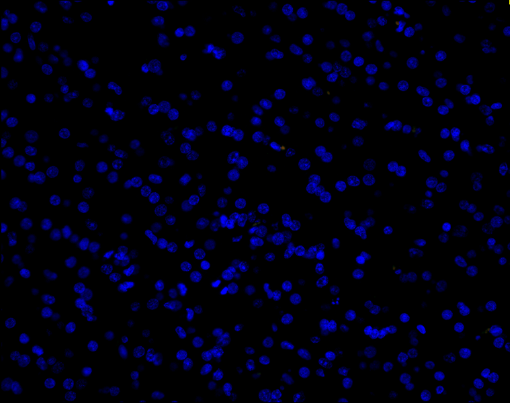

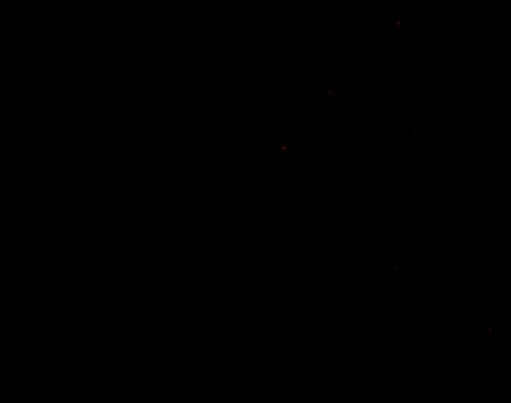

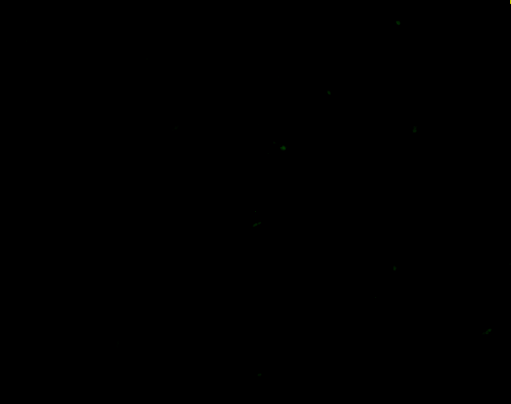

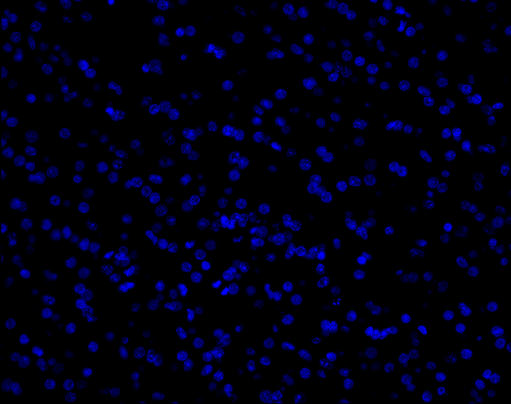


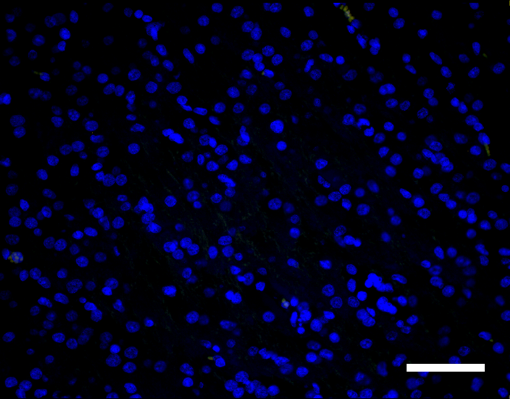


Supplementary figure 2. Negative control. Representative photomicrographs of control*, U.parvum* serovar 3 and 6-exposed subjects that had the target antibodies (anti-GFAP and anti- Iba-1), green and anti-STAT3, red) omitted. Sections were incubated in 1:200 goat anti-mouse-Alexa Fluor 488 and goat anti-rabbit-Alexa Fluor 647 for 3 h at room temperature. Left panel shows immunofluorescent staining of 4ʹ,6-diamidino-2-phenylindole (DAPI, showing cell nuclei, blue). Middle panels show sections without the target antibody did not display non-specific staining. Right panel shows the merged image. Scale bar = 50 µm.
